# Crowdsourcing as an Analytical Method: Metrology of Smartphone Measurements in Heritage Science

**DOI:** 10.1002/anie.201801743

**Published:** 2018-05-28

**Authors:** Rosie Brigham, Josep Grau‐Bové, Anna Rudnicka, May Cassar, Matija Strlic

**Affiliations:** ^1^ Institute for Sustainable Heritage University College London 14 Upper Woburn Place London WC1H 0NN UK

**Keywords:** citizen science, colour analysis, crowdsourcing, heritage science, smartphone

## Abstract

This research assesses the precision, repeatability, and accuracy of crowdsourced scientific measurements, and whether their quality is sufficient to provide usable results. Measurements of colour and area were chosen because of the possibility of producing them with smartphone cameras. The quality of the measurements was estimated experimentally by comparing data contributed by anonymous participants in heritage sites with reference measurements of known accuracy and precision. Participants performed the measurements by taking photographs with their smartphones, from which colour and dimensional data could be extracted. The results indicate that smartphone measurements provided by citizen scientists can be used to measure changes in colour, but that the performance is strongly dependent on the measured colour coordinate. The same method can be used to measure areas when the difference in colour with the neighbouring areas is large enough. These results render the method useful in some heritage science contexts, but higher precision would be desirable.

Crowdsourcing, or more specifically, citizen science projects, defined by Haklay as “the involvement of non‐professional scientists in data collection and, to some extent, its analysis”,^1^ is becoming increasingly popular. By recruiting the help of members of the public to collect and analyse data, physical phenomena can be monitored in a more efficient manner. This type of data collection has been particularly successful in the field of environmental monitoring, where scientific results have been achieved with contributions from untrained users. Participants may note the presence or absence of species,^2^ count specimens,^3^ contribute images of samples for identification,^4^ or help determining the spatial distribution of environmental parameters.^5^ The benefits of crowdsourced measurements are diverse and include increased data‐collection rates, the ability to cover large areas, and the involvement of a wide audience. The widespread availability of smartphones has led many to consider them as a potential analytical instrument,^6^ also in the field of heritage science.^7^, ^8^, ^9^ However, the metrology of this type of measurement has not been explored in the context of crowdsourcing. From an analytical chemistry point of view, a crowdsourced measurement is burdened with a high degree of uncertainty owing to the low degree of control over how a measurement with a smartphone camera (or any other smartphone‐enabled sensor device) is performed. The characteristics of lighting, aspect, resolution, sensor sensitivity, or even image correction algorithms as applied by phone operating systems are all out of the analyst‘s control and need to be taken as contributions towards random uncertainty. Calibration techniques can be applied but a high degree of measurement control may represent a significant engagement barrier. Therefore, in this paper, we explore techniques that potentially require minimum calibration, such as colour and area measurements, that can be taken as proxies for chemical change.^10^, ^11^ Colorimetric analysis of surfaces has been frequently used to evaluate changes in compositions and molecular structures of interest to conservation, and the extensive literature on colour change has been the basis of standards for the storage and display of works of art.^12^ For example, the yellowing of paper is associated with the oxidation of cellulose;^13^ photo‐oxidation results also in the yellowing of many polymers by formation of conjugated unsaturated carbonyl groups;^14^ the pigment known as red lead (triplumbic tetraoxide) fades to white through carbonation and sulfation processes;^15^ and in limestone, glauconite content can be related to a green hue.^16^

This work assesses the precision, accuracy, and intra‐ and inter‐observer repeatability of measurements contributed by visitors using smartphones. Naturally, each of the chemical processes mentioned above and their measurement context have their own requirements in terms of accuracy, but to provide a general criterium of quality, we considered that a successful analytical technique should be able to capture a difference in colour (or area) before it is perceived by the human eye. The human eye can detect colour differences smaller than ΔE=1 under some conditions,^17^ but in practice, this threshold is usually higher. This is expressed by the concept of the just noticeable difference (JND), which has been stablished to vary between ΔE=1 and 3 in different contexts.^18^ For example, when studying colour measurements as an indication of biological growth, Sanmartin and co‐workers^11^ considered colour changes to be imperceptible to humans below ΔE=2. Another possible criterium of quality is defined by the guidelines published by the ASTM,^19^ which define a “lightfast” pigment as one that changes its colour over its lifetime by more than ΔE=8.

The data were collected in two types of experiments: field experiments in heritage sites, where anonymous visitors followed a set of instructions, and controlled laboratory experiments, where measurements were performed by the authors, and the variation of picture quality was simulated by using a diversity of smartphone devices. The purpose of these experiments was to test separately the intra‐observer variability, that is, the precision associated with the performance of commercial smartphone cameras and their operation by a single observer, and the inter‐observer variability, that is, the precision associated with the behaviour of participants. Participants were free to take pictures from any position, with any camera setup, and with any submission frequency. Many of their actions could potentially contribute to measurement uncertainty, including but not limited to the angle of the camera, their position relative to light sources, changes in light conditions, and any post‐processing of the images carried out before submission. These variables were considered as contributions to the uncertainty of the measurement, and no attempt was made to control them to lower the participation barrier and to explore a worst‐case scenario. The experiments focused on two types of measurements, colour and area, which was assessed with a colour‐based method. The colour field experiment was carried out in the Octagon Gallery (University College London, UK) from September 2016 to May 2017. During this time, a sign was installed prompting visitors to collect and submit pictures of an exhibited object (Figure 1 a) via email, Twitter, or Instagram. The sign included a calibration colour chart (Colourchecker Classic, X‐Rite, US), which was used to perform a white balance adjustment of the submitted images. The laboratory colour experiment was performed with six smartphones (iPhone 5S from 2013, Samsung Galaxy XT1063 from 2014, Samsung Galaxy XT1072 from 2015, HTC Desire HD from 2010, Samsung Galaxy Rugby from 2012, and Motorola DEFY from 2010), which were used to take pictures of the same colour chart. A colour reflection spectrodensiometer (X‐Rite 518, X‐Rite, US) was used to obtain reference measurements to be compared with the smartphone measurements. All of the colour measurements were taken in the CIE LAB colour space, and colour differences were calculated by using the CIEDE2000 definition. The images were post‐processed with the open‐source package Fiji for image analysis^20^ to perform a white balance adjustment using the colour chart, and to transform the RGBs in which the submitted images were encoded to the LAB coordinates used in the analysis.

**Figure 1:**
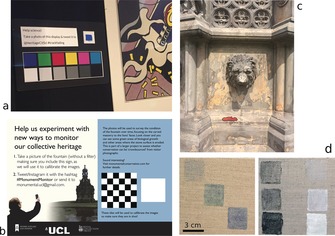
a) Sign used to prompt participants to contribute images in the colour field experiment in the UCL Octagon Gallery. b) Sign used for the same purpose in the area field experiment at the Holyrood Palace Fountain. c) Detail of the Holyrood Fountain, with an area highlighted in red as an example of the type of measurements performed. d) Two samples of colour squares used in the laboratory area experiment. Images (a) and (c) have been contributed by anonymous participants.

The area field experiment (Figure 1 b) was carried out on a stone fountain (Holyrood Palace, Edinburgh, managed by Historic Environment Scotland) from June to September 2017. Similarly, visitors were invited to participate by a sign placed nearby (Figure 1 c), which included, instead of a colour chart, a chequered pattern that could enable the geometrical alignment of images taken from different angles. In the laboratory area experiment, colour squares were used. Each test set comprised three squares with a dark, intermediary, and lighter shade of blue, red, green, and brown as well as black and white (Figure 1 d). Areas of a certain colour were measured using the open source package ImageJ,^20^ following the method proposed in Ref. 21, which is based on the contrast between neighbouring colour areas. The spectral distribution of the light source was measured in all of the instances where it was constant (laboratory experiments and UCL Octagon).

Figure 2 shows the correlation between the coordinates L*, a*, and b* measured with the six smartphones and the colorimeter in the laboratory. It is clear that the L* and b* coordinates display the strongest correlation. Comparisons between devices show a similar trend with all of the tested cameras: The average of the Pearson's correlation coefficient for the a* component measured with each of the phones (0.815±0.07) is significantly lower than the correlation coefficients for the L* (0.963±0.006) and b* (0.915±0.004) components at the 0.05 significance level. The measurements contributed by the visitors in the field experiment provide estimation uncertainties that are very similar to those of the laboratory experiments. In the estimation of L*, the relative standard deviation of the field experiment measurements, studying the aggregate of all images, ranges from 3.3 % in the case of the most precise measurement to 16.5 % for the most imprecise measurement, while this value ranges from 4.7 % to 18.8 % in the case of the laboratory experiments. This indicates that the colour estimates obtained under laboratory conditions, where the only variable is the camera used, are not necessarily more precise than the field measurements where many other factors are not controlled. Therefore, this result suggests that the intra‐observer variability is not higher than the inter‐observer variability, that is, that the behaviour of the participants does not contribute significantly to the overall error, which can be mostly attributed to the quality of the colour capture of the devices. The precision displayed in Figure 2 should be compared with the precision requirements outlined in the introduction: The standard deviation of the measurements displayed in Figure 2 is smaller than the total fading of a lightfast pigment over its lifetime, but larger than the minimum perceptible colour change by the human eye.

**Figure 2:**
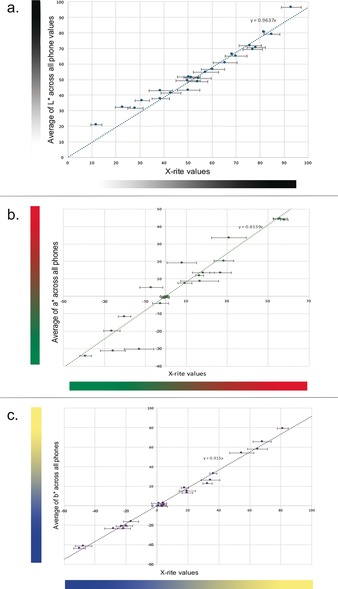
Average A) L*, B) a*, and C) b* values from all phones of colour checker squares compared to X‐Rite control values. L* and b* measurements are consistently nearer to the X‐Rite measured control, represented by the black line, for all devices utilised. The values of a* measured exhibit the lowest precision and accuracy, that is, they are further away from the X‐Rite control line and display larger standard deviations. The vertical error bars are too small to be reported.

Figure 3 displays ΔE values calculated as the difference between the average of all smartphone contributions and the colorimeter measurements. This result is an estimation of the accuracy of the measurement, that is, the distance between the mean of the estimation and the mean of the reference value as measured with the colorimeters. The uncertainty displayed is propagated through the calculation of colour differences following CIEDE2000. These results show that smartphone colorimetry provides potentially useful measurements of colours that have large L* and b* components, but less so when colours have a large a* component (reds and greens). Given this strong dependency of the accuracy on the colour being measured, this technique will be more suited for some practical uses in heritage science than others. For example, Grossi and co‐workers showed that colour change in stone from a variety of different weathering occurred predominantly along the L* and b* axes.^22^ Changes within the L* and b* coordinates on built heritage can also be indicative of biological growth.^10^ Sanmartin and co‐workers showed that within the L*/a*/b* colour space, changes in b* provide an early indication of phototropic colonization.^11^

**Figure 3:**
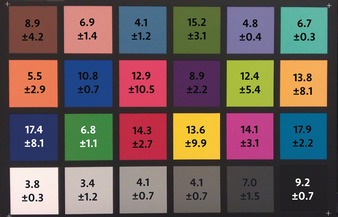
Edited image of the X‐Rite colour chart. The numbers in each square are the values of the total colour difference between the average measurements of colour from every phone compared to X‐Rite measurements. The uncertainty of the measurements indicates the accuracy of the smartphone measurements and corresponds to the standard deviation while the difference indicates their precision.

The method for area detection used here relies on the quality of colour discrimination as it defines an area according to the contrast between its colour and the colour of its surroundings. Furthermore, in this case, the ability of the participants to produce images has an important impact on the usefulness of the raw data. Specifically, while Ref. 21 recommends a minimum resolution of 9 pixels mm^−1^ to perform an analysis, based on their experience with conventional digital photography, the submission from the field experiment with the highest value had a resolution of 0.17 pixels mm^−1^. In the field experiment with the Holyrood fountain, the combination of colour variation between pictures, which in this case were not subjected to a white balance, their different angles, and low resolution in the features of interest led to large uncertainties. For example, the most precise measurement was that of the area highlighted in Figure 1 b, which was measured to be 107±42 cm^2^. This value was obtained by averaging the results extracted from the only five contributions by participants that contained the area of interest. It is not possible to ascertain, using the field experiments alone, which factors (lighting, absence of colour balancing, low resolution of the pictures) contributed more to this uncertainty.

The laboratory experiments, however, provide some insight into how these measurements can be improved. Figure 4 shows the average measurements for all of the coloured squares across all phones. These measurements have been plotted against the value of the difference in colour, ΔE, between the painted square and the background. For the lowest levels of ΔE (the lighter squares), the standard deviation is very large relative to the mean owing to the occurrence of large overestimations. The uncertainty of the measurements is considerably reduced as the difference in colour between the painted square and the background increases. Figure 4 demonstrates that this method produces a good estimate of the area when ΔE is greater than 10. This threshold compares well with some of the colour differences of interest in applications in the heritage field. Grossi and co‐workers found that colour differences on a variety of stone samples left for four months in urban environments subject to sulfation from exposure to SO_2_ ranged from ΔE* values of 5 to 9.^22^ They set a detection level of ΔE* for when discolouration can become noticeable between ΔE*=6–17 using a variety of literature. On average, the ΔE values between the lighter squares and the canvas are between 3 (green) and 9 (blue). These areas proved the hardest to detect. The ΔE of the darkest squares was between 15 (green) and 25 (black). The majority of the measurements on darker areas displayed both a higher precision and accuracy as the values were closer to the true area (700 mm^2^), which was known as it was measured metrically during the preparation of the samples. In the case of the Holyrood fountain, the ΔE values between the area of interest and the neighbouring areas were smaller than the 10 ΔE threshold, which explains the difficulty in estimating the area.

**Figure 4:**
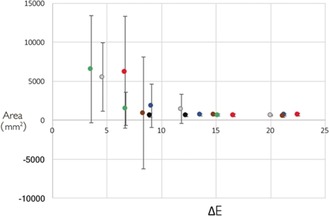
Relationship between the measurement precision and the ΔE* value between the colour of the background and the colour of the painted square. The colours of the points indicate the colour of the painted square. As the colour difference between the background and the painted area becomes more pronounced, the accuracy of the area measurement increases. Error bars represent standard deviations.

These two experiments demonstrate that it is possible to extract colorimetric and area measurements from images contributed by citizen scientists who use their own smartphones, and to quantify their precision and accuracy. The error associated with these measurements is sufficiently small to allow for some useful applications. It is likely that calibration of each of the cameras will improve the estimation of colour and area, but this is a matter for further research. There are other methodological issues that should be independently studied, such as the effect of the level of detail provided to participants, the level of post‐processing of the images and the quality of alignment and white balancing algorithms, the effects of the distance and direction of the photograph, contextual aspects that may influence the number of submissions, as well as methods to analyse large amounts of images efficiently.

## Conflict of interest

The authors declare no conflict of interest.

## Supporting information

As a service to our authors and readers, this journal provides supporting information supplied by the authors. Such materials are peer reviewed and may be re‐organized for online delivery, but are not copy‐edited or typeset. Technical support issues arising from supporting information (other than missing files) should be addressed to the authors.

Supplementary
