# Supplementary material for: Crowdsourcing as an Analytical Method: Metrology of Smartphone Measurements in Heritage Science
Source: Angew Chem Int Ed Engl. 2018 May 28;57(25):7423–7. doi: 10.1002/anie.201801743 (PMC6055710; doi:10.1002/anie.201801743)
Supplement: Supplementary file 1 — Supplementary [file ANIE-57-7423-s001.pdf]

Angewandte Chemie
Eine Zeitschrift der Gesellschaft Deutscher Chemiker

Supporting Information

Crowdsourcing as an Analytical Method: Metrology of Smartphone Measurements in Heritage Science

Rosie Brigham, Josep Grau-Bové, Anna Rudnicka, May Cassar, and Matija Strlic*

anie_201801743_sm_miscellaneous_information.pdf

Contents

1. Method description: Smartphone colourimetry 3
2. Area measurement 3
3. Method description: Holyroodhouse Palace study site 6
4. Data for individual phones 7
5. Smartphone colour calibration....................9
6. Field test area measurements....................16
7. Laboratory area experiment....................21

1. Method description: Smartphone colourimetry

Five colour measurements of each square of an X-Rite ColourChecker Classic colour chart were taken in CIE1976L*a*b* colour space (LAB) by an X-Rite spectrodensiometer (X-Rite 518, X-Rite, US). Images of the chart were then photographed by a variety of phone cameras under the same conditions. The images were then converted into LAB and values taken from each coloured square. These values were then compared to those of the X-Rite. Five different phones were used, the details for each phone can be found in Table 1. They were directly compared against measurements taken by an X-Rite spectrodensiometer.

Table 1: Models of phones used for preliminary investigation

Phone make	Software version	Phone release year	Name
1 iPhone 5s	IOS 10.1.1	2013	iPhone
2 Samsung Galaxy XT1063	Android 4.4.4 KitKat	2014	M5
3 Samsung Galaxy XT1072	Android 5.1.1 Lollipop	2015	CR16
4 HTC Desire HD	Android 2.3.5 Gingerbread	2010	HTC
5 Samsung Galaxy Rugby	Android 2.3.6 Gingerbread	2012	Samsung
6 Motorola DEFY	Android 2.2.2 Gingerbread	2010	Motorola

LAB colour space was chosen as it has a more complex gamut (covers more hues) than RGB and CMYK colour models and can consequently lead to more accurate calculations in colour difference. Within this model, **L*** represents the level of lightness, whereby a value of **L*** = 0 represents black and and **L*** = 100, represents white. Colour channels are represented within the **a*** and **b*** axes. For the **a*** axis, **a*** = -128 corresponds to green and **a*** = 128 corresponds to red. Blue and yellow are conferred by values on the **b*** axis at values of **b***= -128 and **b*** = 128, respectively.

Five measurements for each square of the colour chart were taken using the x-rite, and the mean value for each LAB dimension was calculated. A photograph of the chart was then taken with each mobile phone in similar lighting conditions. The black/white balance of each image was then adjusted using a macro on Fiji. This was done to find a truer value of L*, independent of the lighting conditions in which the images was taken. Then the images were converted to LAB colour space using a similar plugin and five measurements of each dimension were taken for every square. The squares on the colour chart were numbered accordingly and shown in Figure 2.

2. Area measurement

To test the method of analysing areas of colour in submitted images from the study sites, a small preliminary experiment was conducted. This followed a method similar to one used by Thornbrush *et al*, in which a variety of grayscale photographs were taken of buildings in

Oxford. Areas of soiling and blistering were measured using the magic wand tool in Adobe Photoshop with a tolerance setting of 50 out of 255 (20%), with Contiguous selected to eliminate non-adjacent pixels.

Table 2. Models and specifications of devices used for taking images for preliminary investigation

Device model	Pixels per mm in test images	Focal length (mm)	Maximum camera resolution (pixels)	Year released
iPad Pro (10.5 inch)	8.50	3.99	4032 x 3024	2017
iPhone 6	10.32	4.15	3264 x 2448	2014
iPhone 6s	12.45	4.15	4032 x 3024	2015
Motorola E2	0.45	Unknown	2560 X 1920	2015
Samsung GT-I9300	9.01	3.7	3264 x 2448	2012
ZTE B2017G	6.623	3.7	4608 x 3456	2016
Cannon DSLR 1000D	17.13	5.6	3888 x 2592	2008

For our investigation, squares of colour of differing intensity were painted onto pale brown canvas. Each square measured roughly 200 x 300 mm and the colour coverage was not completely uniform due to fact they were painted. This was intentional as the colour of biological growth is also often not completely uniform. Photographs of each different board were then taken using a variety of mobile phones and at slightly different angles with the same lighting conditions. Photographs were also taken with a Cannon 1000D digital single lens reflex camera (DSLR) for comparison. The details for each phone can be found in Table 2; unfortunately, they are different the ones from the preliminary test, as these were not available at the time.

The perspective in the images were rectified and straightened using the ‘distort’ tool in Adobe Photoshop CS6. The scale of each image was set using the known width of the canvas boards (100 mm) using Fiji (version 1.0). This process meant that the images did not need to be re-sized to the same dimensions. The white balance was adjusted by applying three new curve adjustment layers and configuring each to the lightest, darkest and 50% grey point respectively, with the lightest point being selected from a white tile. A single white tile instead of a greyscale was used to assess how well white balance can be rectified across different images with few calibration targets. This approach was used instead of the macro approach utilised in the previous investigation as grayscale calibration was unavailable.

The area of the colour was measured in Fiji, an open source code for image processing, using the Magic Wand tool, which detects areas based on the colour similarity between neighbouring pixels, with a tolerance of 31 out of 255 and then repeated with a tolerance of 20 in both colour and 8-bit grayscale. This was chosen instead of the 50 out of 255 used by Thornbrush, as the average colour difference within each square was ~ΔE* 7, which represents a difference of roughly 30 shades within the 8-bit images. The captured areas were then measured and compared against each other, to determine how effective different mobile phone cameras with differing levels of image quality were at capturing areas of colour.

3. Method description: Holyroodhouse Palace study site

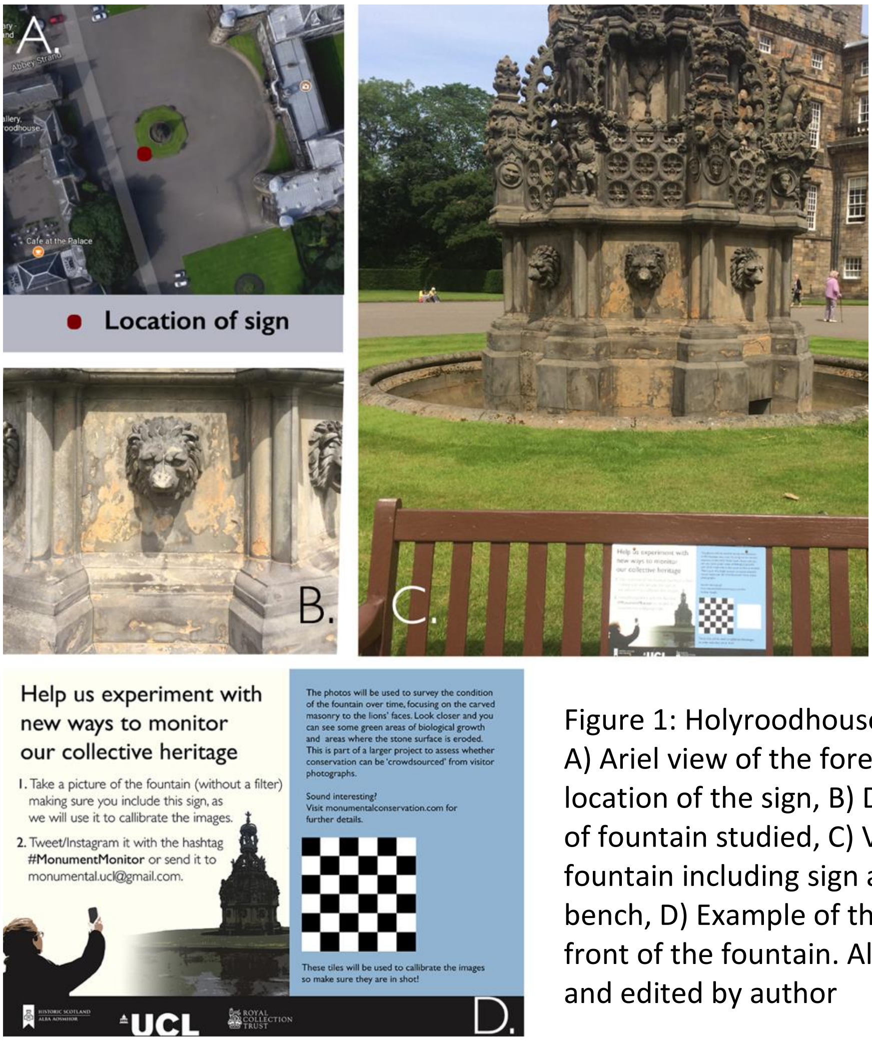

Figure 1: Holyroodhouse Palace Fountain. A) Ariel view of the forecourt with location of the sign, B) Detail of section of fountain studied, C) View of the fountain including sign attached to bench, D) Example of the sign placed in front of the fountain. All images taken and edited by author

Situated in the middle of Edinburgh, Holyrood Palace is one of the premier tourist destinations of the city receiving around 250,000 visitors per year. The fountain, (Figure 1) is situated in the middle of the forecourt at the visitor entrance and thus receives a significant amount of footfall. As an impressive monument, it is often the subject of pictures as visitors enter the palace, which makes it an ideal site for this experiment.

The fountain is constructed out of binny sandstone and is prone to biological colonisation, notably of moss, lichen and algae. Algon treatment has been used as a way to counteract this however biofilms often develop quickly after treatment, especially under overhanging elements . A report into the treatment used measurements of lightness to measure the change on the fountain over time, however it is difficult to take ongoing measurements accurately as access to the uppermost parts of the fountain requires scaffolding. The upper section of the fountain is also prone to weed growth which, despite posing little threat to the stone, detracts from the appearance of the fountain. These plants are often not possible to reach from the ground and so have to be sprayed with an herbicide. Visitors were asked to take photographs of the bottom south-east facing side of the monument over the experimental period.

4. Data for individual phones

Figure 2 shows the number code that corresponds to each square of the colour chart. The graphs in Figure 3 compare the mean values of the measurements for each LAB dimension across the first twelve colour squares. The values for the x-rite have been included for comparison against each of the sample mobile phones. A visual colour reference for each LAB dimension is provided to the left the graphs and the colours of the squares are shown below. Due to the low variance between repeated measurements of the same colour, error bars have been omitted.

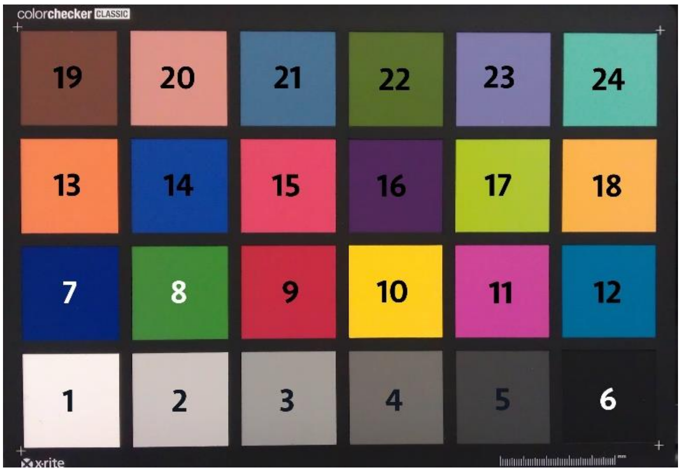

Figure 2: Colour chart used for measurements with reference numbers added

The values across the charts are similar which indicate the devices record colour on a similar level. However, there is a clear difference between some of the phones. For values of L* the Motorola consistently records values above the x-Rite except for the darkest shades of grey; the CR16 records values just below the x-Rite whilst the iPhone and M5 seem to register the closest values more consistently. Interestingly, the darkest shades produce the greatest variance, especially for square six and seven.

Squares one to six are a greyscale and thus should produce very low values for both a* and b*. Although the x-rite registers a small value for square one on the b* graph, it is curious that the phones, in general, register more colour, especially towards the yellow. This could be due to the lighting conditions in which the photographs were taken or to the white balancing that each image underwent before transformation to LAB. However further tests would be needed in different light conditions, with and without the balancing script in order to ascertain whether this was the cause.

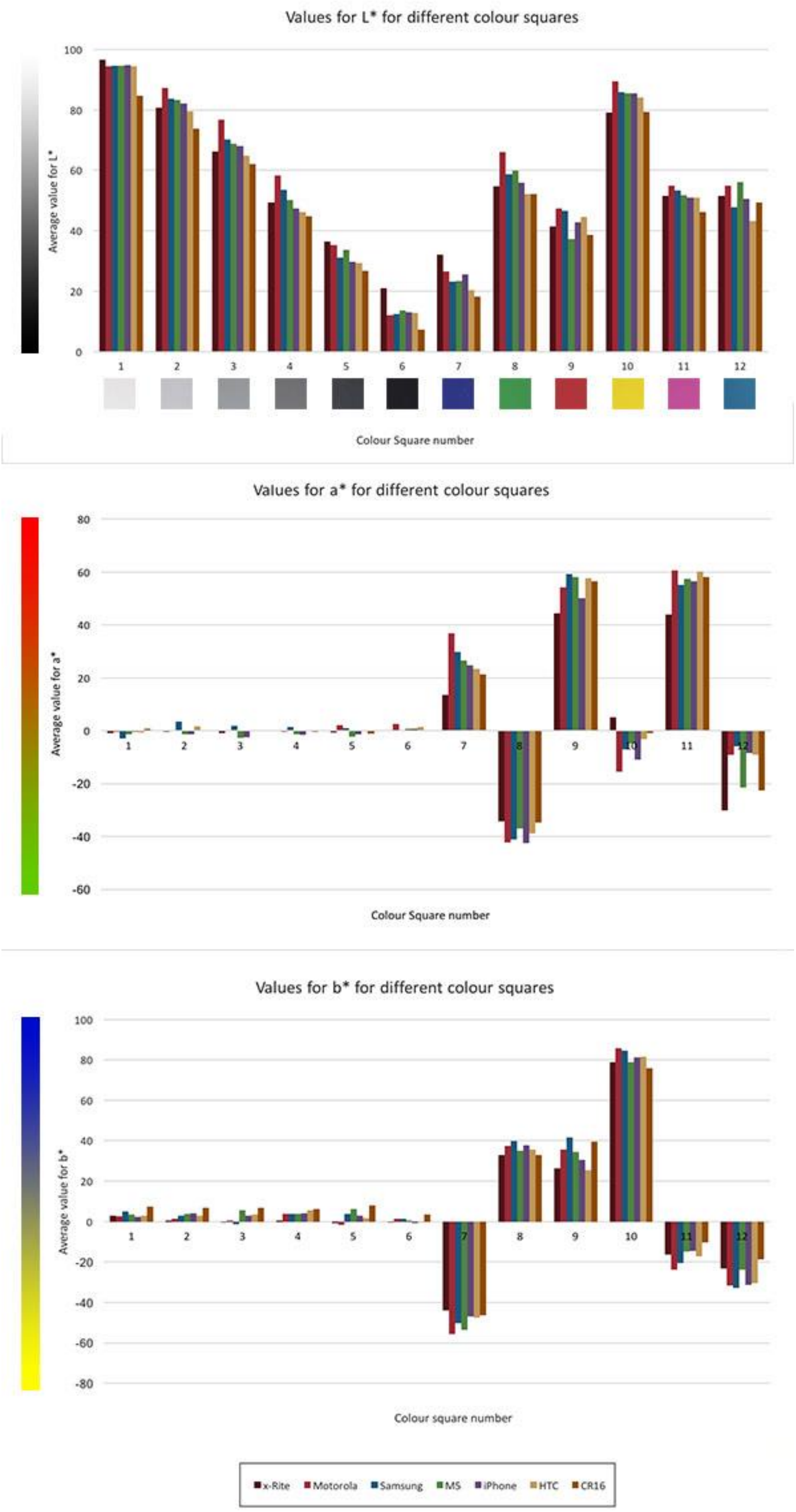

Figure 3: Comparison of x-Rite and phone mean LAB values across first 12 colour squares

5. Smartphone colour calibration

The estimations of colour produced with pictures from each smartphone are improved with a calibration with measurements of known L*a*b values, here called “Reference” measurements. The relationship between the smartphone measurements and reference measurements is linear for a* and b* coordinates, and exponential for the L* coordinate in each case. Figures 4 to 9 display the calibration curves for the three coordinates for each of the tested phones. Table 3 summarizes the calibration equations.

Table 3: Results of the linear and exponential regression when x-rite colour chart measurements are plotted against each phones measurements

	Motorola	Samsung	M5	iPhone	HTC	CR16
**L***	$y = 21.71e^{0.0171x}$ $R^2 = 0.966$	$y = 19.928e^{0.0169x}$ $R^2 = 0.964$	$y = 20.242e^{0.0163x}$ $R^2 = 0.962$	$y = 20.77e^{0.0165x}$ $R^2 = 0.964$	$y = 0.8203x + 10.965$ $R^2 = 0.948$	$y = 21.71e^{0.0171x}$ $R^2 = 0.966$
**a***	$y = 0.7286x + 0.3009$ $R^2 = 0.759$	$y = 0.8353x - 2.4125$ $R^2 = 0.880$	$y = 0.8306x - 0.1591$ $R^2 = 0.937$	$y = 0.7928x - 0.3826$ $R^2 = 0.888$	$y = 0.8023x - 1.773$ $R^2 = 0.929$	$y = 0.8471x - 0.728$ $R^2 = 0.956$
**b***	$y = 0.8901x + 1.6631$ $R^2 = 0.981$	$y = 0.8356x - 1.3018$ $R^2 = 0.981$	$y = 0.946x - 1.5605$ $R^2 = 0.984$	$y = 0.908x - 0.5757$ $R^2 = 0.990$	$y = 0.9409x + 0.0009$ $R^2 = 0.977$	$y = 1.0006x - 4.9029$ $R^2 = 0.983$

Figure 4: Calibration for the three colour coordinates for the Motorola phone

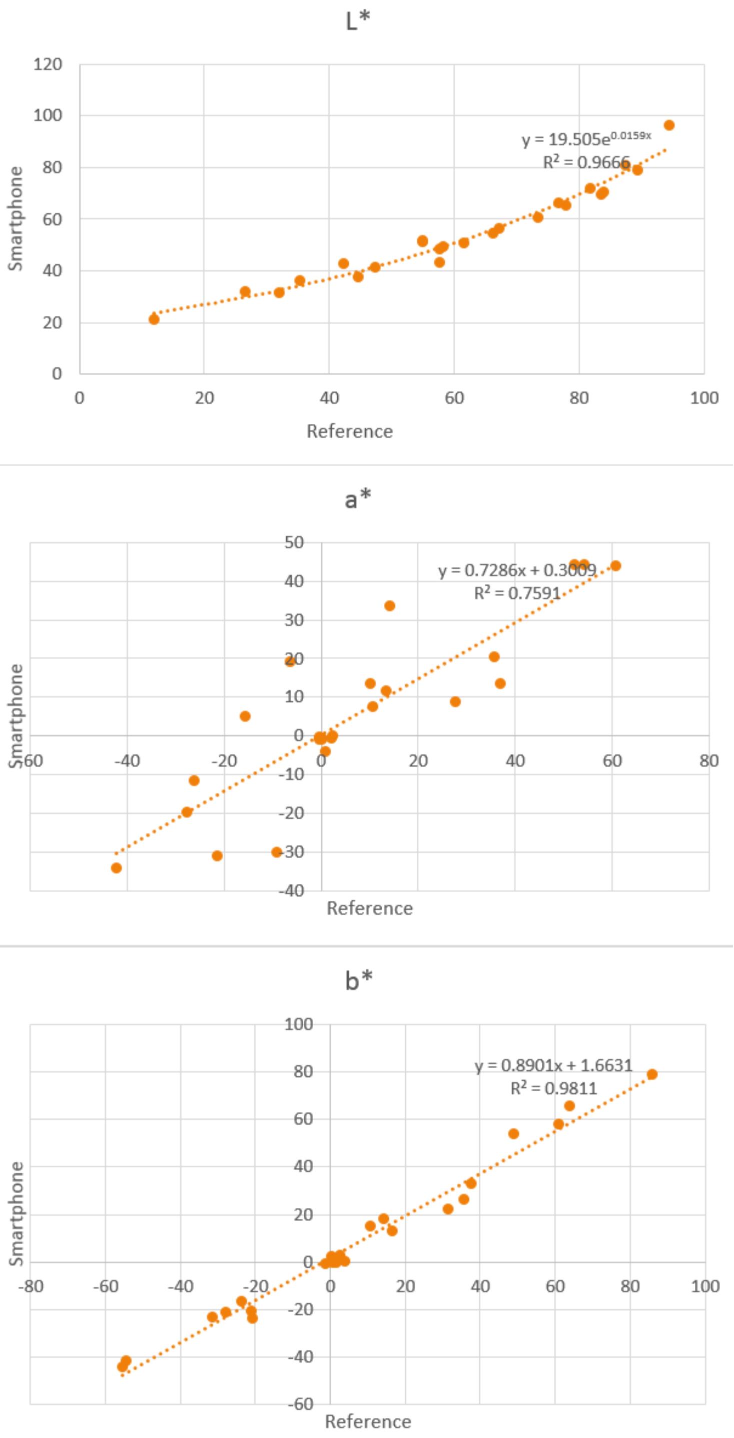

Figure 4: Calibration for the three colour coordinates for the Samsung phone

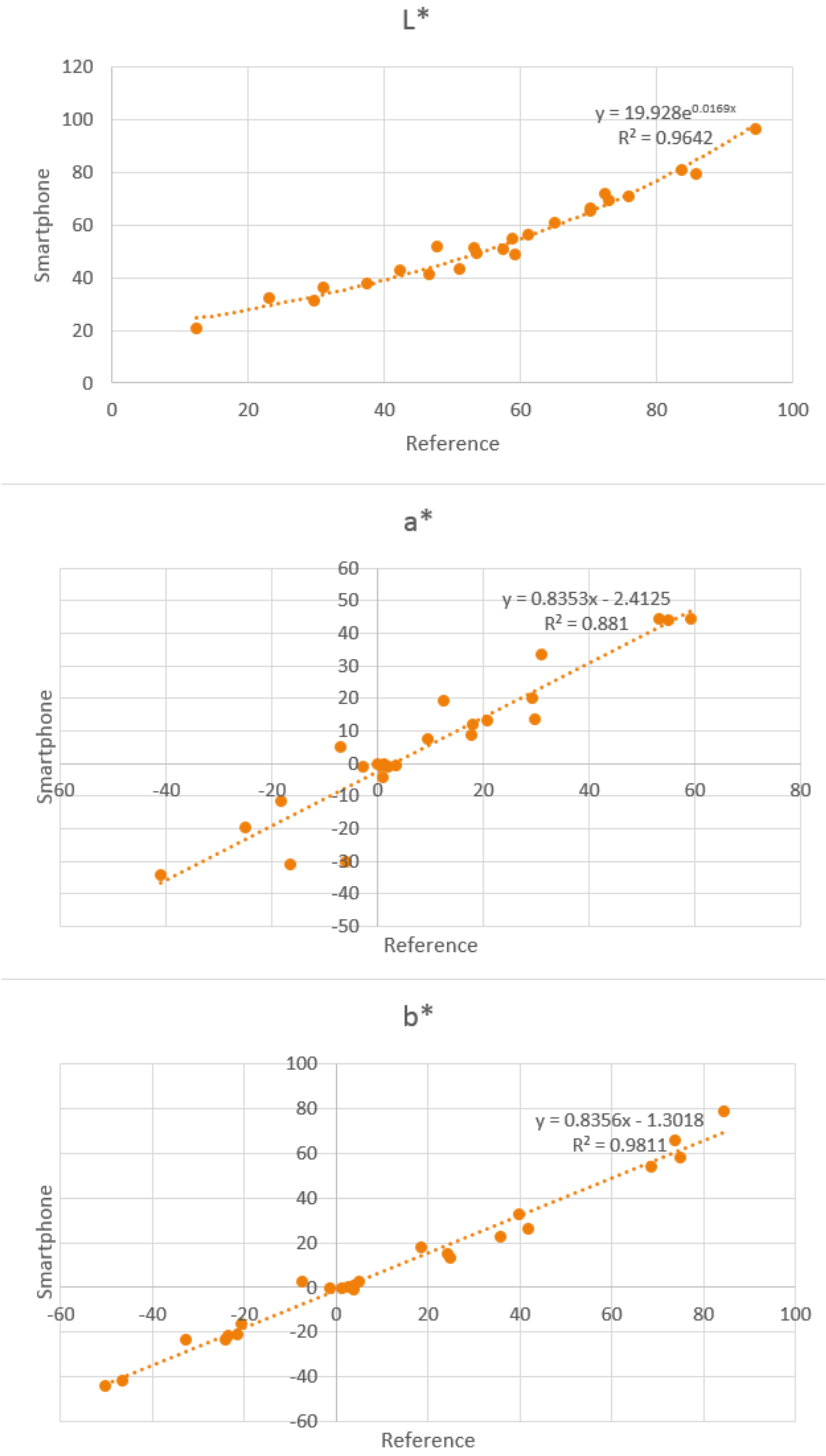

Figure 6: Calibration for the three colour coordinates for the M5 phone

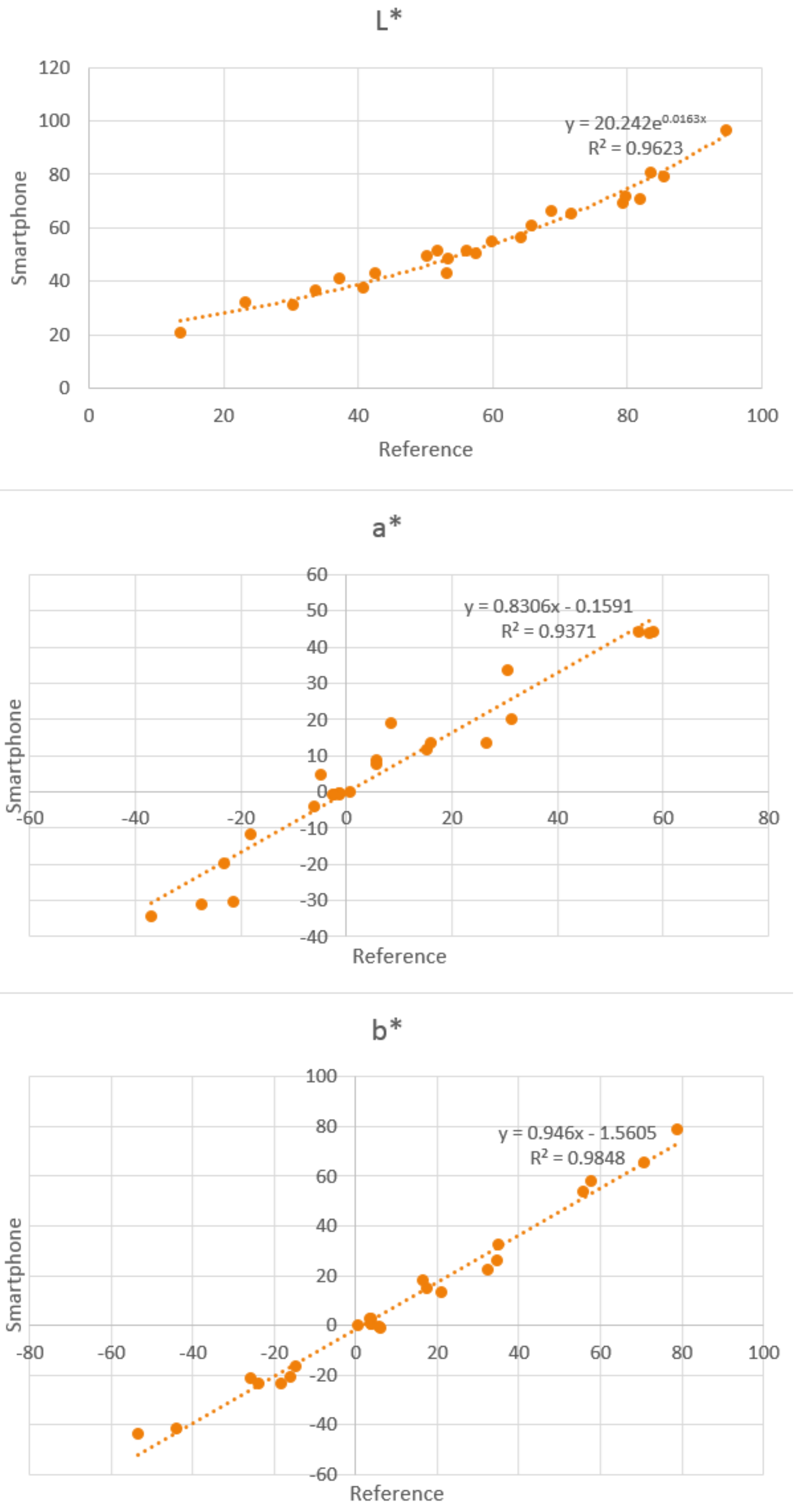

Figure 7: Calibration for the three colour coordinates for the iPhone

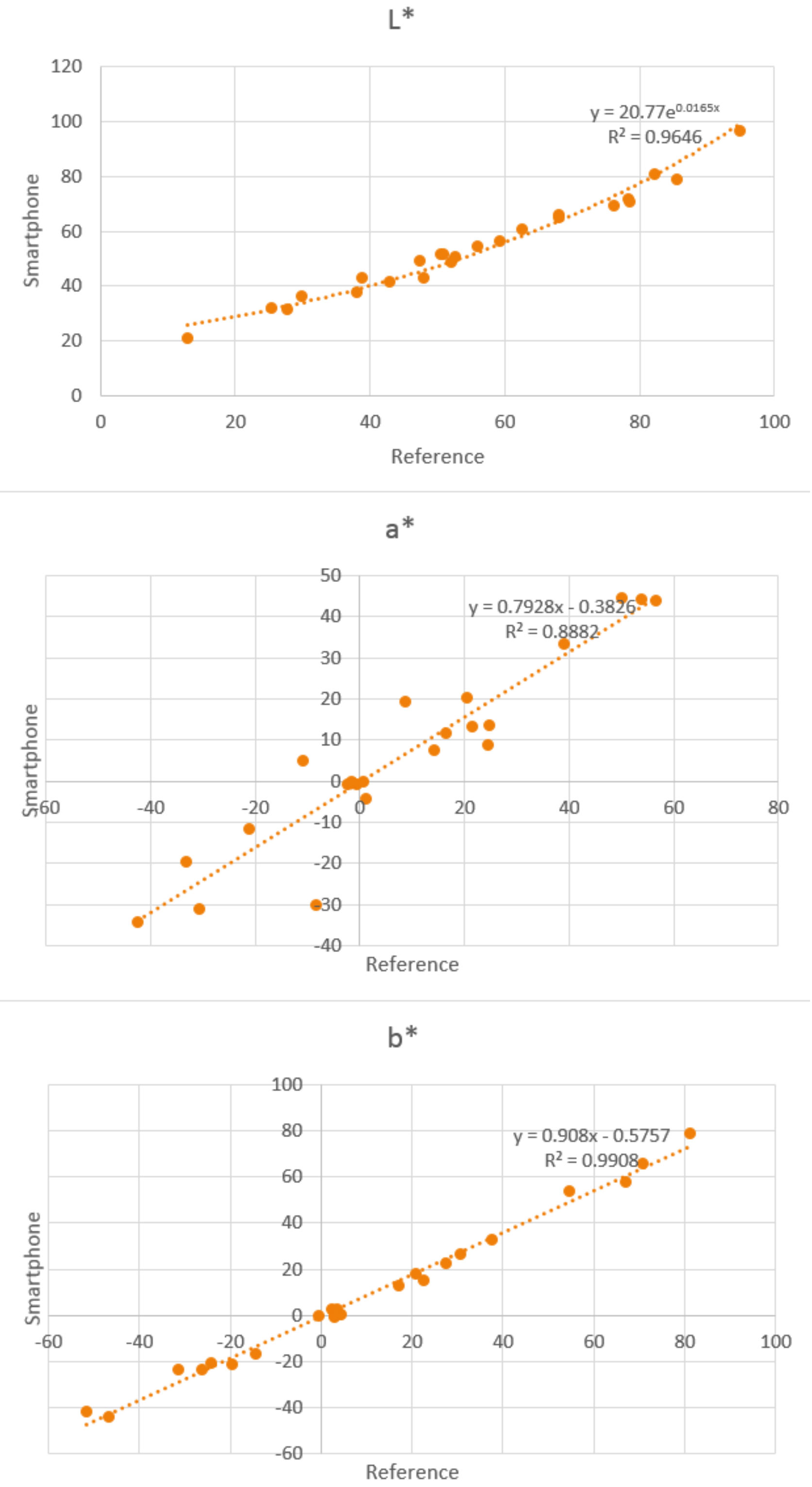

Figure 8: Calibration for the three colour coordinates for the HTC phone

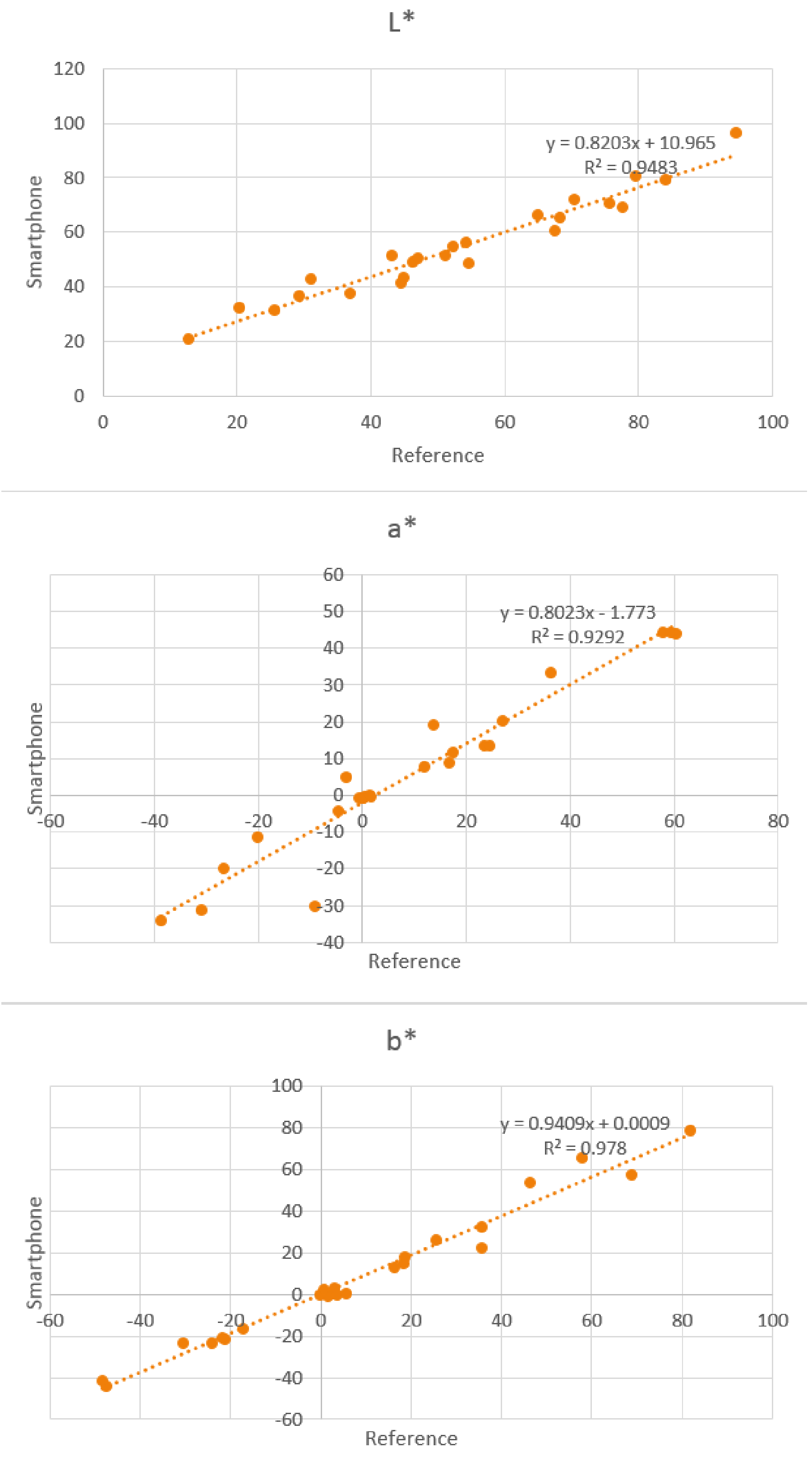

Figure 9: Calibration for the three colour coordinates for the CR16 phone

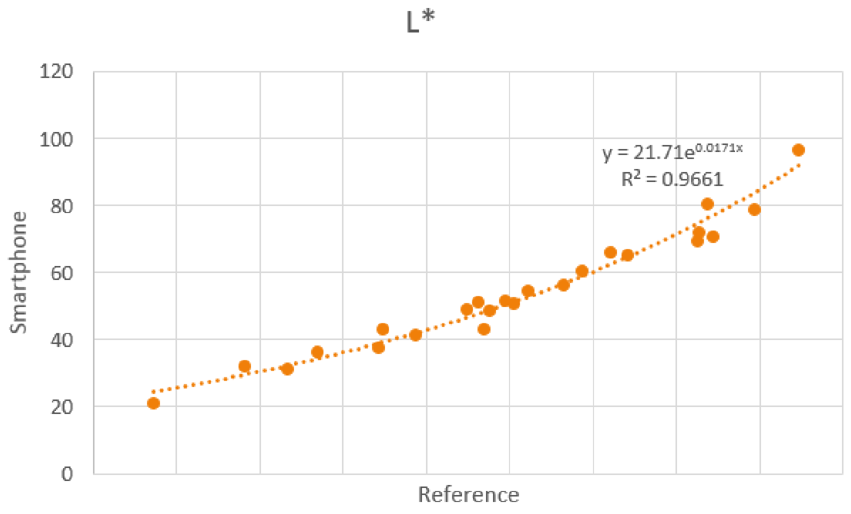

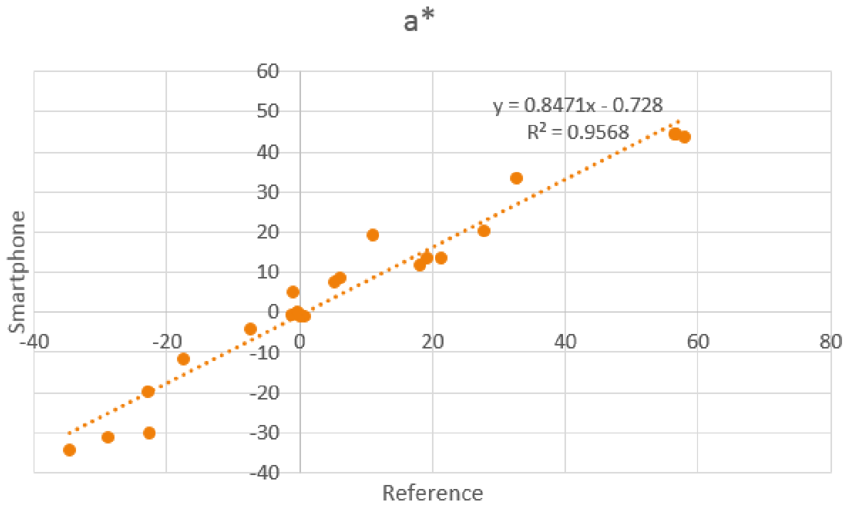

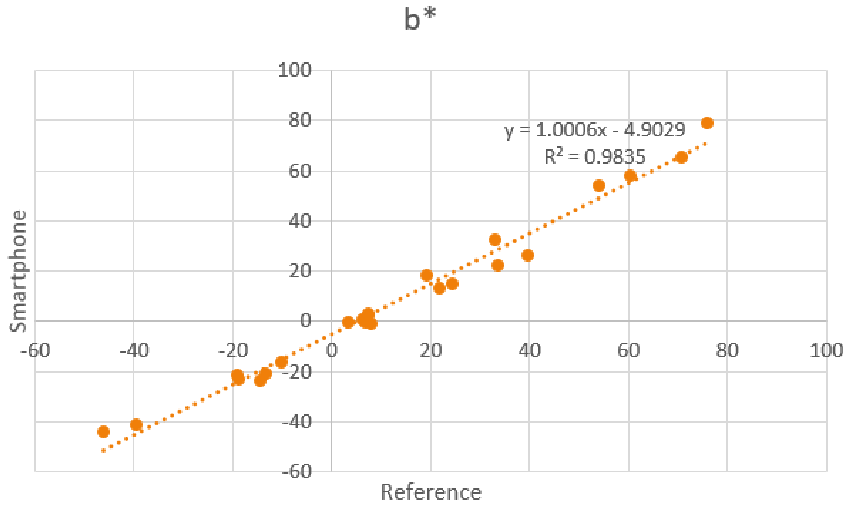

6. Field test area measurements

A total of 21 images were submitted of the Holyrood fountain over the period of the experiment; nine of those were uploaded via Twitter, five via Instagram and seven were submitted via email. Although each image captured the same subject, they varied greatly in lighting and weather conditions. Of the submitted images, 15 were deemed useful in that they captured the area of the fountain that is being studied. Images in where there was heavy rain could not be used because the raindrops vastly decreased the quality of the image. The five largest images were then used to try and measure areas of colour to identify areas of discolouration or delamination.

Table 3 lists examples of the five photos in which the quality was good enough for analysis. In each case, the image has been calibrated in the manner described above and the magic wand tool used to select areas of discolouration around the lion’s head. The same point of the fountain was used in each image for the selection. In columns where there is no image, the selection was extended to the boundaries of the shape and was too large to be included.

Table 4: Images used for colour area analysis of Holyrood Fountain

Date	Image with highlighted areas	Area to the left of lion’s head	Area underneath lion	Area to the right of lion head
23/06	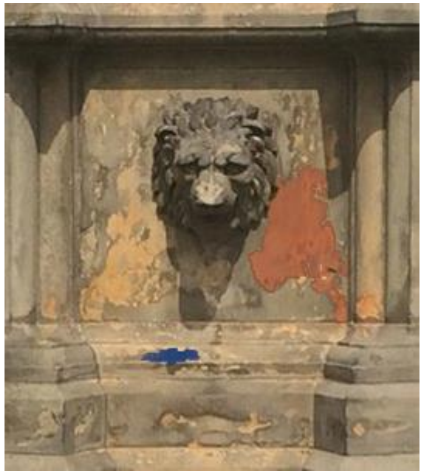	No useful measurement	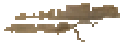 12171 mm^2 (σ 4089 mm^2)	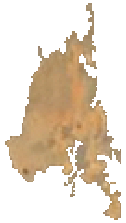 90800 mm^2 (σ 9942 mm^2)
25/06	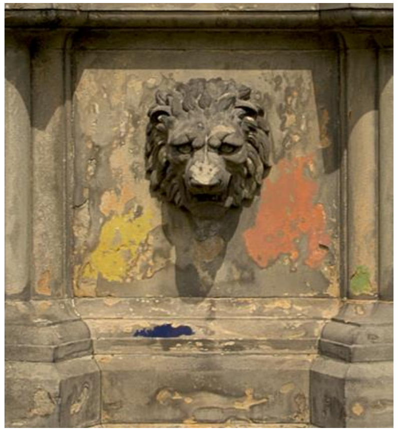	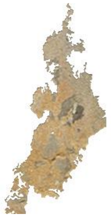 74953 mm^2 (σ 3742 mm^2)	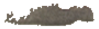 7167 mm^2 (σ 799 mm^2)	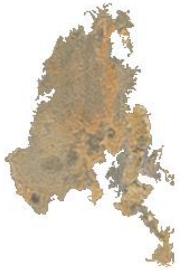 87031 mm^2 (σ 10538 mm^2)

11/07	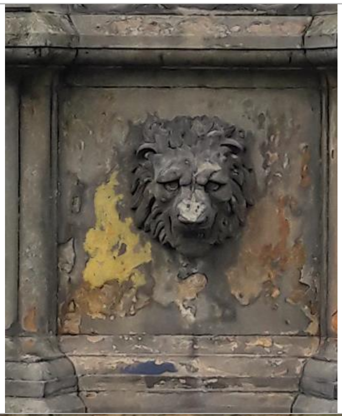	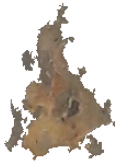<br>57120 mm^2<br>(σ 7470 mm^2)	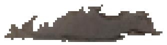<br>7647 mm^2<br>(σ 784 mm^2)	No useful measurement
11/07	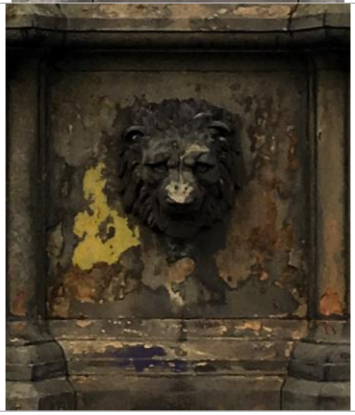	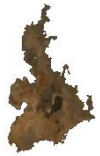<br>45610 mm^2<br>(σ 6316 mm^2)	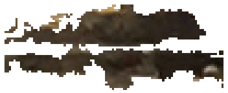<br>16170 mm^2<br>(σ 4689 mm^2)	No useful measurement

15/07	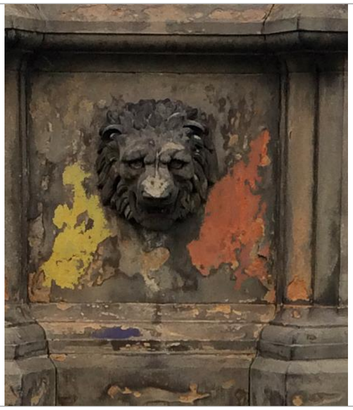	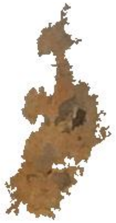 60218 mm^2 (σ 3712 mm^2)	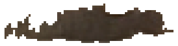 6044 mm^2 (σ 176 mm^2)	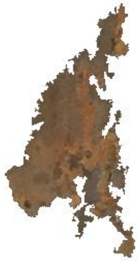 90366 mm^2 (σ 6720.4 mm^2)

This table gives a clear indication as to the ability to measure areas of colour using this method. The scale is considerably smaller than those in the preliminary experiment in which a pixel/mm density was recommended to be above 9. In the submitted images, the largest image was 0.17 pixel/mm. Although the shapes of the selected areas are similar, there is a clear discrepancy across them. The calculated area measurements from these images include considerable outliers. The area measured underneath the lion (coloured blue in the reference images) range from 6044 mm^2 to 16170 mm^2. Similarly, the left area measured (coloured orange in the reference images) range from 45610 mm^2 to 74953 mm^2. Each of these images were taken within a relatively short period of time, therefore it would be unexpected to see be much discolouration or growth in areas of discolouration. However, the selected areas of the measurement and the area measured are different for each one

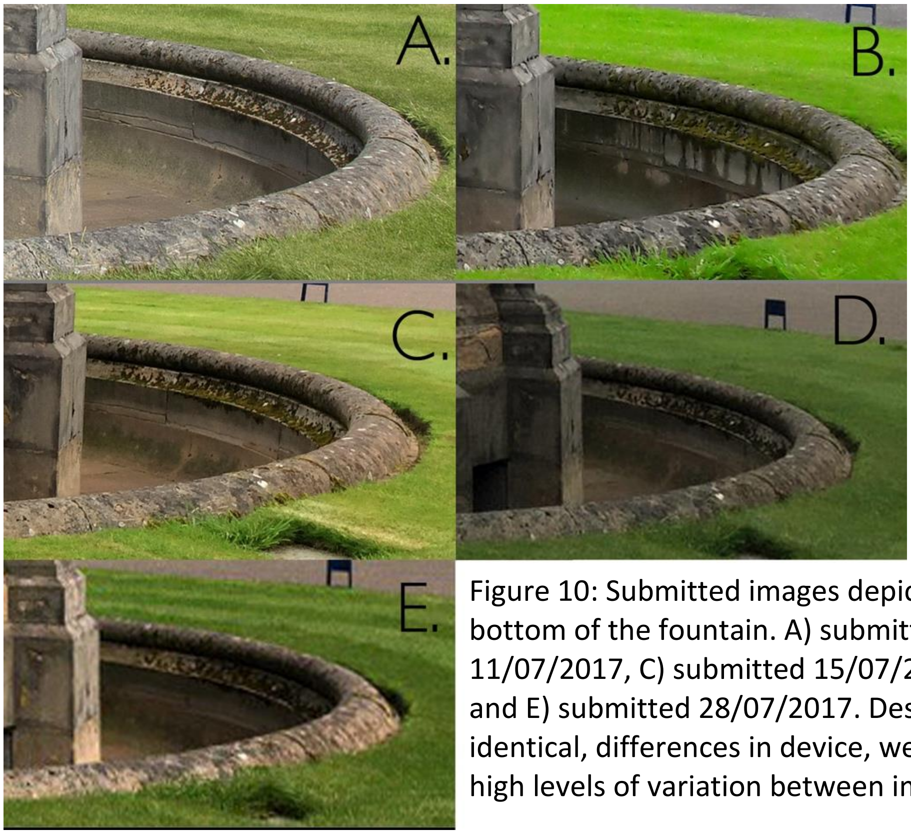

Figure 10: Submitted images depicting algae growth at the bottom of the fountain. A) submitted 26/06/2017, B) submitted 11/07/2017, C) submitted 15/07/2017, D) submitted 26/07/2017 and E) submitted 28/07/2017. Despite the image location being identical, differences in device, weather and lighting result in high levels of variation between images.

The limited level of calibration could explain these discrepancies. Figure 8 focuses on an area of algae at the bottom of the fountain. The cast, contrast and colour balance across each of these photos are visibly very different, and portray the intensity of algae growth in different ways, despite there being no actual difference in algae growth over the course of the experiment.

Discrepancies in these images can be identified by analysing the calculated ΔE* values between time points, (see Figure 11). If the images were accurate and comparable to each other, the

values would be near identical; however, a wide range between approximately 2 and 10 is seen, with over half of the measurements exceeding 6.7. Importantly, these latter values lie within a range of 5.5 - 17.28, indicating that there has been discolouration of the stone, even though there has not.

The variability of ΔE* values, often culminating in an overestimation, suggests that unless a robust colour calibration system is in place, use of crowdsourced images in this fashion could be inherently unreliable.

	A	B	C	D	E
A		8.4	5.2	9.9	10.1
B	8.4		6.7	3.2	5.1
C	5.2	6.7		8.7	8.4
D	9.9	3.2	8.7		2.7
E	10.1	5.1	8.4	2.7	

Figure 11: ΔE* between LAB values taken from the same point at the bottom of the fountain from the 5 images in figure 8. The LAB colour has been included as a reference next to the letter designations. The colour formatting of the table cells signifies how close the values are to the ΔE* of the two x-rite measurements; dark green indicates that the value is higher, light green indicates the value is lower.
Although, the LAB colour measurements are taken at the same point, the values are clearly different, resulting in a range of delta E values between them and a high degree of variability.

7. Laboratory area experiment

To assess the ability to measure areas of colour within an image, multiple phone cameras were used to photograph a series of colour graded squares of known area. Each test set comprised of 3 squares with a dark shade, intermediary and lighter shade of blue, red, green and brown as well as black and white. All squares measured 700 mm^2. The detectable area was measured using the magic wand selection tool in Fiji, set at threshold limits of 20 and 31. The final calculated area was determined as the average of 3 individual analyses for each dataset.

The results for all the different coloured squares for each different selection method can be seen in Appendix 1. Regardless of threshold, the magic wand tool was able to more accurately calculate the area of each coloured square in images acquired by cameras taken at a higher resolution (pixel density ≥ 9 mm^2). For images with lower resolution, the wand tool would often select the entire surface of the canvas which increased the average and the standard error. However, the tool seems to deliver very varied results across the areas of colour. Often by moving the wand tool by a single pixel, and sometimes not at all, the tool would deliver a different measurement. Although this was most common on the lighter squares and images with lower resolution, it was a pattern found with all the squares which contributed to the high levels of error.

However, the lightest squares were most consistently overestimated, in particular the red, green and brown squares. The red, for example, was overestimated in every photograph, with 6550 mm^2 being the smallest and 17,451 mm^2 the largest measurement (taken from the iPad Pro and Motorola images respectively).

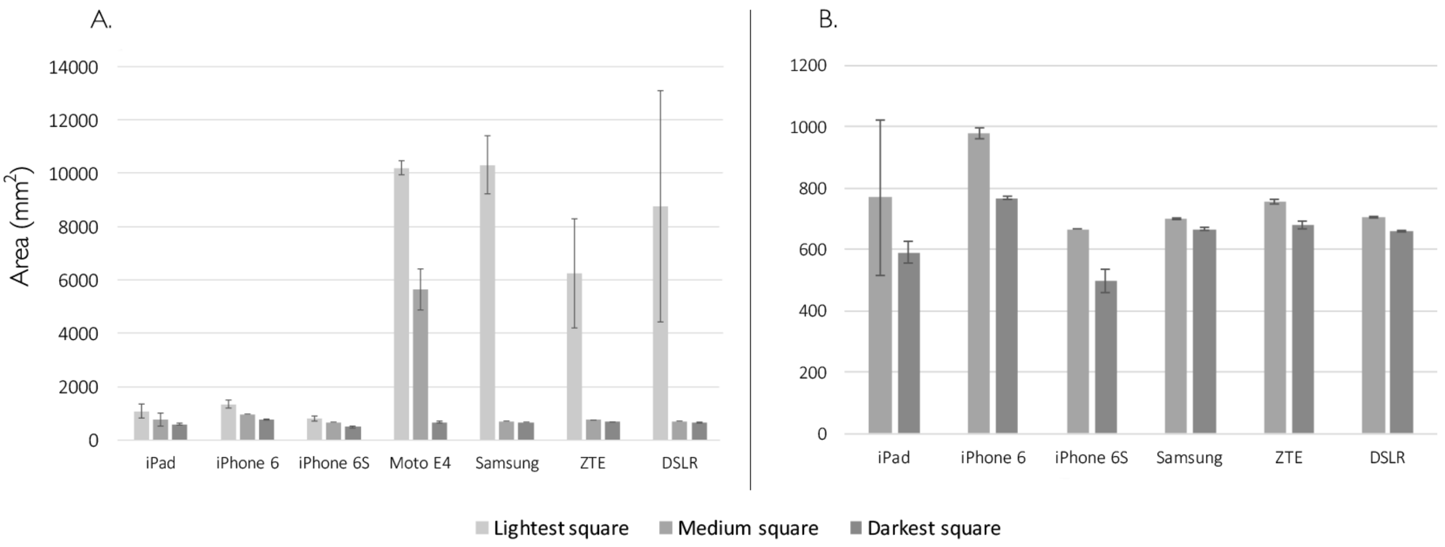

Figure 13: Average measured area of painted white squares, taken from colour images using a tolerance of 20. A) All results B) Results with lightest square and Moto E4 removed, to aid in data visualisation. Actual area of each square is 700 mm^2. All devices overestimated the area of the light grey square, particularly the Moto E4, Samsung, ZTE and DSLR. Accuracy increased with the measurements of the medium grey and dark grey squares. Error bars represent standard deviation, n = 3.

Figure 13 demonstrates the difference in graphs when the outlying results are omitted. In 6a measurements of the lightest square were overestimated by over a factor of ten by 4 of the 7 cameras which has distorted the chart. The results for the camera with the lowest resolution (the Motorola) and the lightest squares have been omitted in 6b, which renders the remaining results more comparable. This figure shows that measurements of the darker squares are more effective at measuring the area of colour. The measurements are closer to 700 mm^2 and the error bars are smaller. However, considering every square measured 700mm^2 there is still a degree of inaccuracy. For example, there is a difference of 311 mm^2 for measurements of the medium square between the iPhone 6 and iPhone 6S. This represents a difference of nearly 50% of the area being measured. The omission of the extreme results from the lightest squares and Motorola have been repeated across all the results and can be seen in Figures 15 and 16.

Figure 14 shows the average measurements for all the coloured squares across all phones. These measurements have been plotted against the value for the change in colour, or Delta E (ΔE^*), between the painted square and the canvas. For the lowest levels of ΔE^*(the lightest squares) the error bars are extremely large though they shrink considerably as the difference in colour between the painted square and the canvas increases. These charts demonstrate that

this is a poor method of calculating areas of colour when the difference between the area being measured and the background is less that ΔE* 10. Figure 14b shows the same graph but with the smaller values of ΔE* removed. Here it is clear that this method is more useful, and the error is approximately ±200 mm^2.

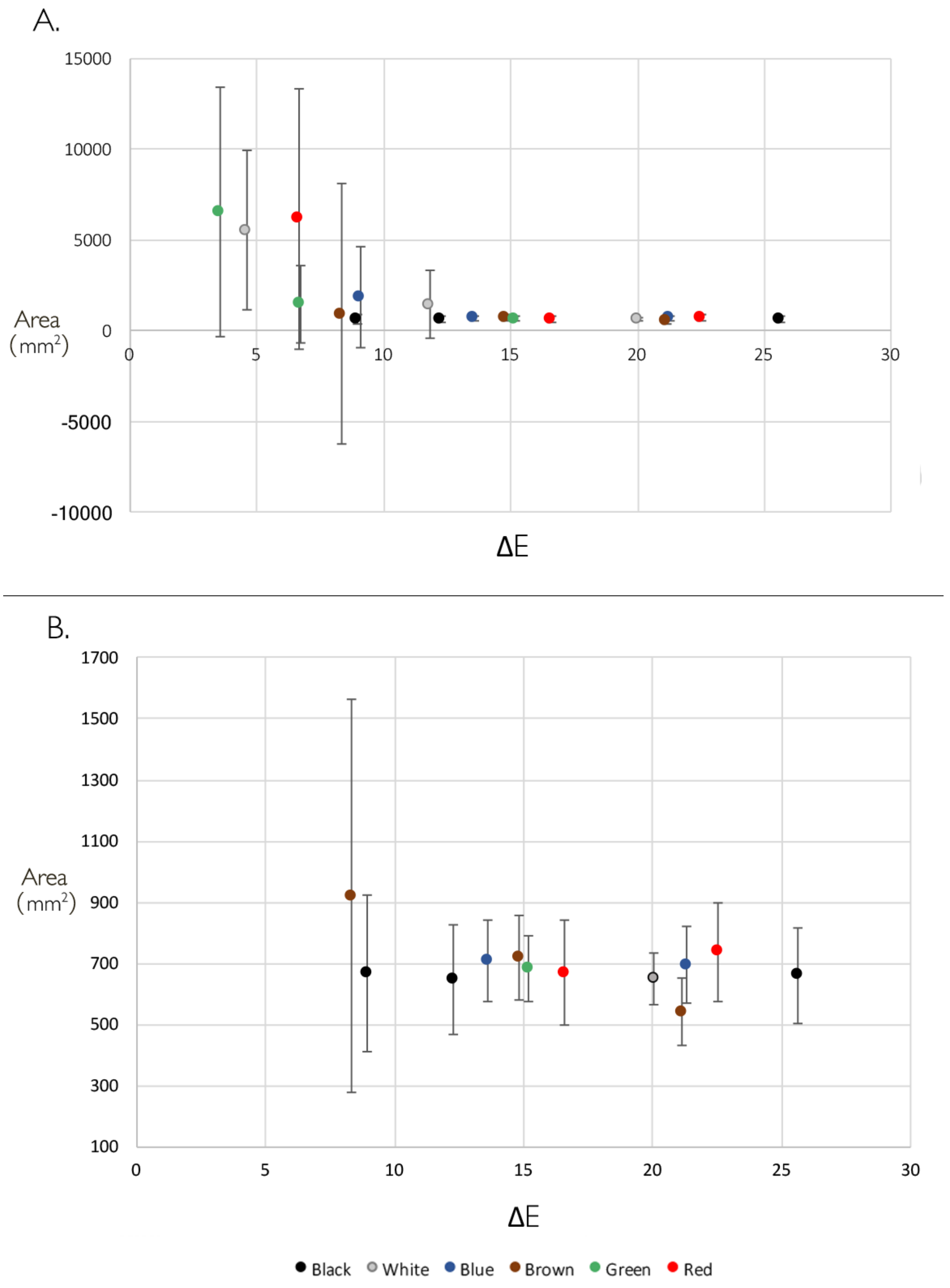

Figure 14: Averages of all measurements of areas of colour, taken from all test images, compared against the ΔE* between the colour of the canvas, and the colour of the painted square. The ΔE* measurement was calculated by using two colour measurements taken by an X-Rite spectrodensitometer. A) As the colour change between the canvas and the painted area becomes more pronounced, the accuracy of measuring the area increases. B) as A), with the outlier values removed to aid in data visualisation. Error bars represent standard deviation, n = 21, with 3 per device.

The area of the darker squares was more accurately determined using the lower threshold of 20. However, conclusions regarding the use of RGB or grayscale images for analysis were not able to be ascertained from this dataset. In certain instances, the determined area of the squares was more accurate if the image was provided as grayscale as per analysis of the brown squares, in which the measurements were closer to 700 mm^2 and the margin of error smaller, particularly from the iPhone 6**.** However, in others, such as for the green squares, the margin of error was larger when calculated from the greyscale image. Thornbrush *et al.* found that it was sometimes difficult to distinguish between soiling caused by airborne pollutants, organic growths and other blackening such as water staining when using grayscale images to analyse pollution on stone buildings. They suggest that colour images provide more information.

Figure 15: Complete results for second preliminary experiment in which squares were painted onto a canvas and photographs taken with a variety of cameras. After this, the Magic Tool wand was used to detect the area of each colour. The bars represent the areas of colour measured from the different images taken from the different phones. The different coloured graphs represent the different colour squares that were measured, black, white, blue, brown, green red respectively from top to bottom. The three different columns show the different results from the different selection techniques, using a tolerance of 31 with the magic wand, 20 with a coloured image and 20 with a greyscale image.

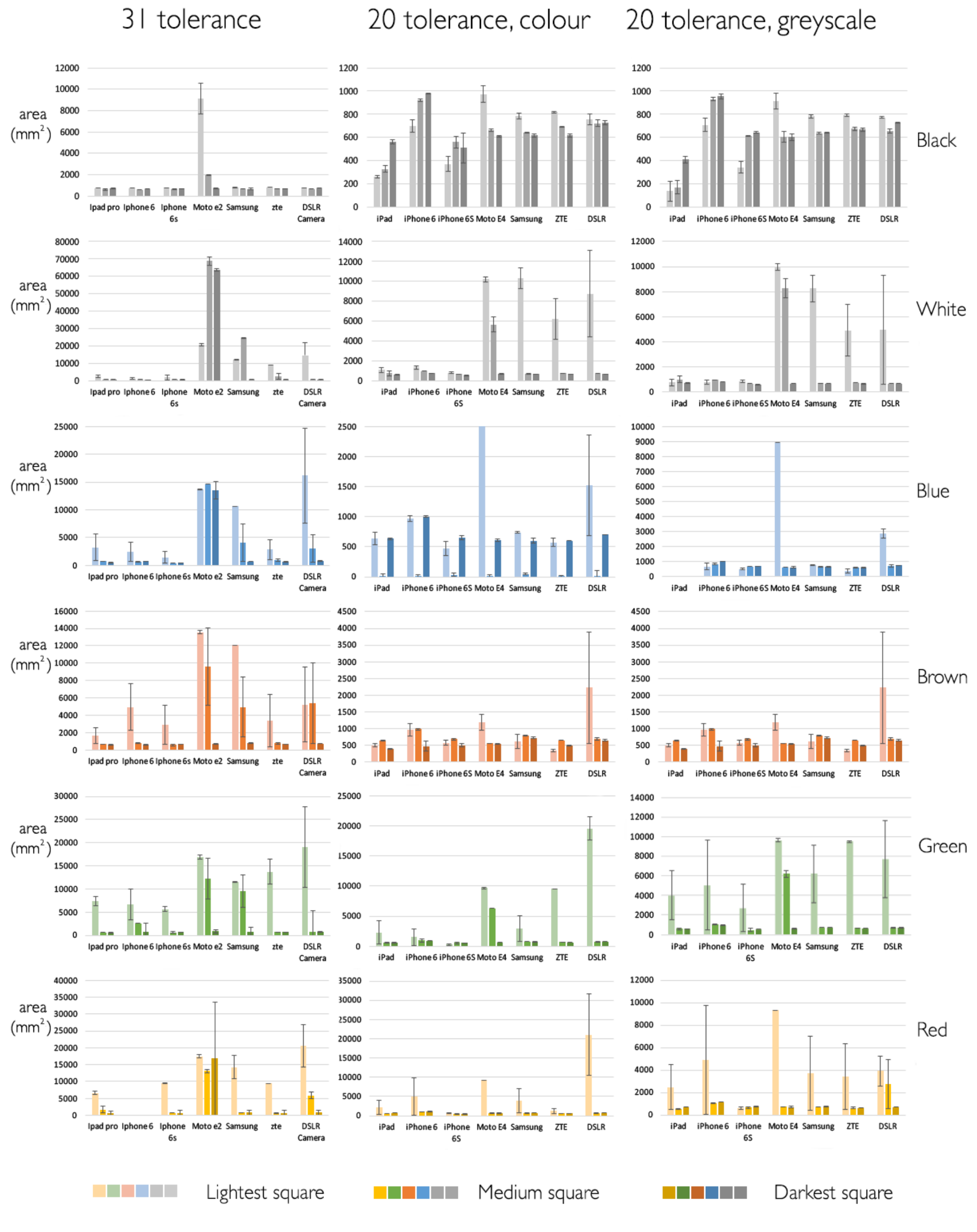

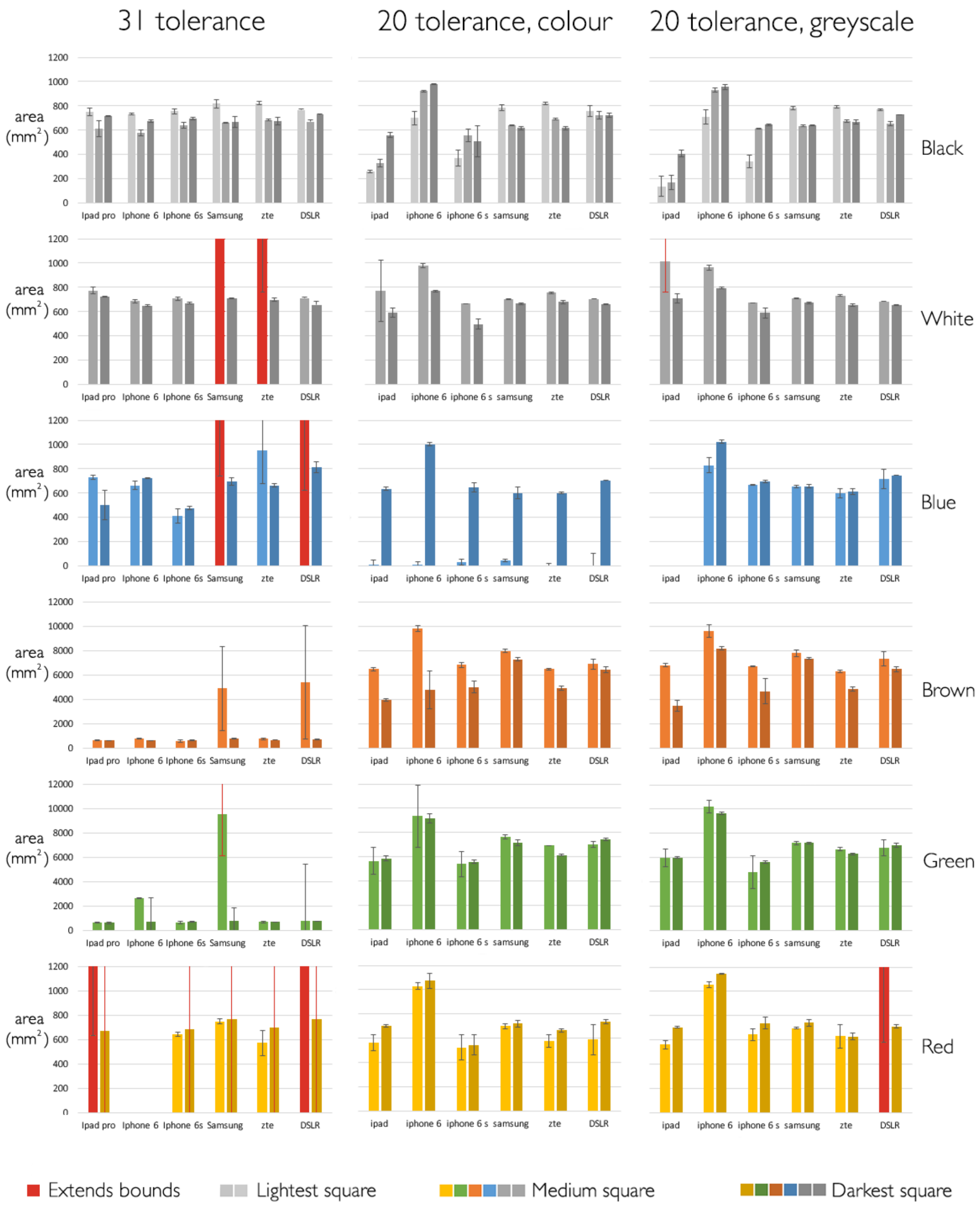

Figure 16: As Figure 15, however the results for the lightest squares and the Motor E2 phone have been removed. Additionally, each of the y axis have been restrained to 1200 mm^2. This is for ease of data visualisation and comparison across different measuring methods. Any result that extends these bounds have been highlighted in red and can be seen in full in A1.
